# Low Plasma Lecithin: Cholesterol Acyltransferase (LCAT) Concentration Predicts Chronic Kidney Disease

**DOI:** 10.3390/jcm9072289

**Published:** 2020-07-18

**Authors:** Andrea Baragetti, Alice Ossoli, Arianna Strazzella, Sara Simonelli, Ivano Baragetti, Liliana Grigore, Fabio Pellegatta, Alberico L. Catapano, Giuseppe Danilo Norata, Laura Calabresi

**Affiliations:** 1 Dipartimento di Scienze Farmacologiche e Biomolecolari, Università degli Studi di Milano, 20133 Milano, Italy; andrea.baragetti@unimi.it; 2 Centro E. Grossi Paoletti, Dipartimento di Scienze Farmacologiche e Biomolecolari, Università Degli Studi di Milano, 20133 Milano, Italy; alice.ossoli@unimi.it (A.O.); arianna.strazzella@unimi.it (A.S.); sara.simonelli@unimi.it (S.S.); 3 Department of Nephrology and Dialysis, Ospedale Bassini, ASST Nord Milano-Cinisello Balsamo, 20092 Milano, Italy; ivano.baragetti@asst-nordmilano.it; 4 S.I.S.A. Centro per lo Studio della Aterosclerosi, Ospedale Bassini, Cinisello Balsamo, 20092 Milano, Italy; grigore.centroatero@gmail.com (L.G.); fabio.pellegatta@guest.unimi.it (F.P.); 5 IRCCS Ospedale Multimedica, Sesto San Giovanni, 20099 Milano, Italy

**Keywords:** lecithin:cholesterol acyltransferase, chronic kidney disease, high-density lipoproteins

## Abstract

Low high-density lipoprotein-cholesterol (HDL-c) is the most remarkable lipid trait both in mild-to-moderate chronic kidney disease (CKD) patients as well as in advanced renal disease stages, and we have previously shown that reduced lecithin:cholesterol acyltransferase (LCAT) concentration is a major determinant of the low HDL phenotype. In the present study, we test the hypothesis that reduced LCAT concentration in CKD contributes to the progression of renal damage. The study includes two cohorts of subjects selected from the PLIC study: a cohort of 164 patients with CKD (NefroPLIC cohort) and a cohort of 164 subjects selected from the PLIC participants with a basal estimated glomerular filtration rate (eGFR) > 60 mL/min/1.73 m^2^ (PLIC cohort). When the NefroPLIC patients were categorized according to the LCAT concentration, patients in the 1st tertile showed the highest event rate at follow-up with an event hazard ratio significantly higher compared to the 3rd LCAT tertile. Moreover, in the PLIC cohort, subjects in the 1st LCAT tertile showed a significantly faster impairment of kidney function compared to subjects in the 3rd LCAT tertile. Serum from subjects in the 1st LCAT tertile promoted a higher reactive oxygen species (ROS) production in renal cells compared to serum from subjects in the third LCAT tertile, and this effect was contrasted by pre-incubation with recombinant human LCAT (rhLCAT). The present study shows that reduced plasma LCAT concentration predicts CKD progression over time in patients with renal dysfunction, and, even more striking, it predicts the impairment of kidney function in the general population.

## 1. Introduction

Chronic kidney disease (CKD) has a major public health impact all over the world, due to its systemic nature [1] and the link with multiple risk factors [2]. The risk of hospitalization and overall mortality increases with the decrease of the renal function and the increase in albuminuria, a sign of advanced renal damage typical of diabetics [3]. While traditional risk factors (diabetes, hypertension, and hypercholesterolemia) contribute to the elevated cardiovascular risk at early CKD stages, inflammation, malnutrition, loss of body weight, muscular mass, and cachexia become more dominant during advanced stages and predict the mortality rate. In this “reverse epidemiology” context, low cholesterol levels determine the highest rate of cardiovascular morbidity and mortality [4,5].

We have previously demonstrated that reduced high-density lipoprotein (HDL) cholesterol is the most remarkable lipid trait both in mild-to-moderate CKD patients [6] and in hemodialysis patients [7]. These initial observations were later confirmed in different populations [8,9]. The uremic and inflammatory status are known to alter HDL lipid and protein composition; HDL from CKD patients present with reduced levels of apolipoprotein (apo)A-I, apoA-II, lecithin:cholesterol acyltransferase (LCAT) and paraoxonase, while pro-oxidant factors such as malondialdehyde, myeloperoxidase, and symmetric dimethylarginine are increased [7,10,11,12]. CKD also affects the HDL subclass distribution; patients with CKD show an increased content of pre-beta HDL (preβ-HDL), owing to the reduced LCAT activity [7]. All these modifications are believed to compromise some key HDL functions, such as the ability to promote cholesterol efflux [11,13,14] and to promote endothelial nitric oxide production [12] and also hamper the anti-inflammatory properties [15]. Interestingly, the HDL profile observed in CKD patients mirrors the profile found in carriers of genetic LCAT deficiency [16], possibly due to the acquired LCAT deficiency in CKD [7]. Since the alterations in the lipid/lipoprotein profile in LCAT-deficient carriers are involved in the pathogenesis of renal disease [17], which represents the first cause of morbidity and mortality in these subjects [18], we test the hypothesis whether the reduced LCAT concentration in CKD contributes to the progression of renal damage. Moreover, we investigate whether low plasma LCAT concentration predicts CKD incidence and evolution in the general population. Finally, we test in vitro the impact of serum from subjects with low plasma LCAT concentration on oxidative stress in podocytes and tubular cells.

## 2. Materials and Methods

### 2.1. Study Cohorts

The study included two subsets of subjects selected from the PLIC study [6,19,20,21], an epidemiological and prospective large survey of the general population of the northern area of Milan conducted at the Center for the Study of Atherosclerosis, Bassini Hospital (Cinisello Balsamo, Milan, Italy). The study was approved by the Ethic Committee of the University of Milano (SEFAP/Pr.0003).

The first cohort included 164 patients with CKD at the basal visit (the NefroPLIC cohort). This population has been previously described in detail [6,22]. Briefly, CKD patients were enrolled through the outpatient ambulatory of the Unit of Nephrology and Dialysis of Bassini Hospital and followed-up for 84 months. Patients with any of the following conditions: inflammatory or infectious disorders, congenital or hereditary kidney diseases, previous dialysis (either peritoneal or hemodialysis), glomerulonephritis, cardiovascular events in the previous six months, microvascular complications (either as retinopathy, neuropathy, foot ulcers or gangrenes), previous major cardiac or vascular surgery, malignant neoplasms, and thyroid dysfunction were excluded from the study.

The second cohort consisted of 164 subjects randomly selected from the PLIC cohort with comparable age, relative prevalence of men and women, and with a median basal estimated glomerular filtration rate (eGFR) of 67.51 (60.01 as minimum and 122.03 as maximum) ml/min/1.73 m^2^.

All subjects provided a written informed consent and all procedures were performed in accordance with the Declaration of Helsinki.

### 2.2. Study Endpoints

eGFR was estimated using the Cockroft–Gault formula. For the NefroPLIC cohort, the estimation of the Modification of Diet in Renal Disease equation was derived as well, in accordance with validated guidelines for CKD classification [23]. For the basal characterization of the cohorts and to study correlation with clinical parameters, both groups were divided by LCAT concentration tertiles. To evaluate CKD progression, we considered dialysis entry (either peritoneal or hemodialysis) and/or basal creatinine level doubling as outcomes over time in the NefroPLIC cohort (up to seven years as maximum follow-up). In order to verify whether reduced LCAT concentration is a specific marker of the progression of renal disease, independently of other major co-morbidities, we also assessed the predictive value of reduced LCAT concentration for the following end-points occurring during follow-up: (1) all-cause mortality (from medical registries); (2) cardiovascular fatal or non-fatal events (either as atrial fibrillation, heart failure, stroke, transient ischemic attack, coronary-artery by-pass grafting, peripheral revascularization, amputations for gangrenes or microvascular complications); (3) composite outcome of dialysis entry and/or creatinine doubling + total mortality; (4) composite outcome of dialysis entry and/or creatinine doubling + cardiovascular fatal or non-fatal events; (5) composite outcome of dialysis entry and/or creatinine doubling + cardiovascular fatal or non-fatal events + all-cause mortality.

In parallel, to study the relation between the LCAT concentration and CKD incidence in the PLIC cohort, annual eGFR changes (annual variation of mL/min/1.73 m^2^) and eGFR decline below 60 mL/min were considered as end-points.

### 2.3. Biochemistry

Blood and urine samples were collected after overnight fasting. Plasma, serum, and urine samples were stored at –80 °C until analysis. Lipid/apolipoprotein profile, liver enzymes, and cardio-metabolic markers were determined as previously described [6]. Plasma LCAT concentration was determined by an immunoenzymatic assay developed in our laboratory [16]. Pre-beta HDL content was determined by 2-D electrophoresis [24].

First morning urine samples from three different days were collected, and the urinary albumin was measured using an immunoturbidimetric assay (Cobas Mira Plus Analyzer, Roche, France). The mean value of the three samples was calculated and normalized relative to the urinary levels of creatinine according to the guidelines of the American Diabetes Association. Urine samples were cultured at baseline to exclude infection as a possible etiology of proteinuria.

### 2.4. In Vitro Studies

Experiments were performed using two different renal cell lines: immortalized human podocytes, maintained in Dulbecco′s Modified Eagle Medium (DMEM) containing 1g/L of glucose supplemented with 10% fetal bovine serum (FBS), 1% L-Glutamine, and 1% antibiotics; immortalized human tubular cells (HK2), cultured in DMEM enriched of nutrient mixture F-12 with 10% FBS, 1% L-Glutamine and 1% antibiotics.

To evaluate reactive oxygen species (ROS) production, both podocytes and tubular cells were starved in serum-free medium for 2 h and then incubated for 30 min with HEPES buffer containing 5 µM of carboxy-H2DCFDA (Molecular Probes, Invitrogen, Thermo Fisher, Rockford, IL, USA) before 1-h incubation with media containing 2% v/v of serum. Oxidation of the probe was detected by monitoring fluorescence at 517–527 nm. Data were normalized on total protein content measured with microBCA assay (Thermo Fisher Scientific, Rockford, IL, USA) on cell lysate.

Podocin gene expression was evaluated by quantitative real time polymerase chain reaction (RT-PCR). Briefly, RNA was isolated and transcribed to cDNA with the iScriptTM cDNA synthesis kit (BioRad, Hercules, CA, USA). Gene expression analysis was performed using Sso Advanced Universal SYBR Green Supermix (BioRad, Hercules, CA, USA) and the expression was normalized to a housekeeping gene (β-actin).

To test the effect of LCAT in determining the serum ability to promote ROS production, sera from 1st LCAT tertile subjects (*n* = 11) were incubated at 37 °C for 6 h with rhLCAT [24] in the amount required to reach the final LCAT concentration of 6 μg/mL (identical to the average LCAT concentration in 3rd LCAT percentile subjects) or with the same volume of saline. Lipids and apolipoproteins were evaluated before and after incubation, as described above. After incubation, ROS production induced by sera was evaluated, as described above, in both cell lines. The effect of serum-free rhLCAT (6 μg/mL) was also tested.

### 2.5. Statistical Analyses

Statistical analyses were performed using SPSS^®^ v.23.0 for Windows^®^ (IBM Corporation^®^, Chicago IL, USA) software. A Shapiro–Wilk test was performed to verify the normal distribution of continuous variables. For normally distributed variables, the mean ± standard deviation (SD) is reported. The t-test was used for comparisons; for non-normally distributed variables, the median and inter-quartile range (IQR, as the range between the 25th and the 75th percentile around each median value) are reported, and the Mann-Whitney U nonparametric test was performed. For dichotomous variables, the chi squared test and relative risk (95% C.I., confidence interval) assessment were performed. Multiple stepwise Cox-regression models were used to verify in the nefroPLIC cohort the predictive value of reduced HDL-c or LCAT concentration for the studied outcomes (the forward selection of co-variates was applied with entry testing (set at 0.05) and removal testing (set at 0.1) based on the probability of the Wald statistic). Kaplan–Meier survival curves and log-rank tests compared CKD progression rates by LCAT concentration tertiles and they were compared by both univariate log-rank test and Cox-regression models setting the 3rd tertile (high LCAT concentration) as a reference vs. the 2nd and 1st tertile (lower LCAT concentration). A linear regression model was used to analyze the predictive value of reduced HDL-c or LCAT concentration (as continuous variables) for eGFR change over follow-up in the PLIC cohort. T-statistics were derived from linear regression models to estimate the size of the difference relative to the variation in HDL-c or LCAT concentration. eGFR changes across LCAT tertiles were compared, adjusting for co-variates, by the Analysis of Co-Variances (ANCOVA) model. Non-normally distributed variables were log-transformed when included in the multivariable models. The test of collinearity was implemented for each multivariable regression model to derive the Variation Inflation Factor (VIF) and excluded redundant covariates.

Box plots for data distributions, reporting mean values with 10th to 90th upper and lower bounds, were generated using GraphPad Prism 5^®^ for Windows^®^ (Graphpad Software Inc.^®^, La Jolla, CA, USA).

Results of the in vitro studies were compared by the Kruskall–Wallis test.

For all analyses, *p*-values < 0.05 were considered statistically significant.

## 3. Results

### 3.1. Lower Plasma LCAT Concentration Predicts Disease Progression in Patients with Chronic Kidney Disease

**Figure 1:**
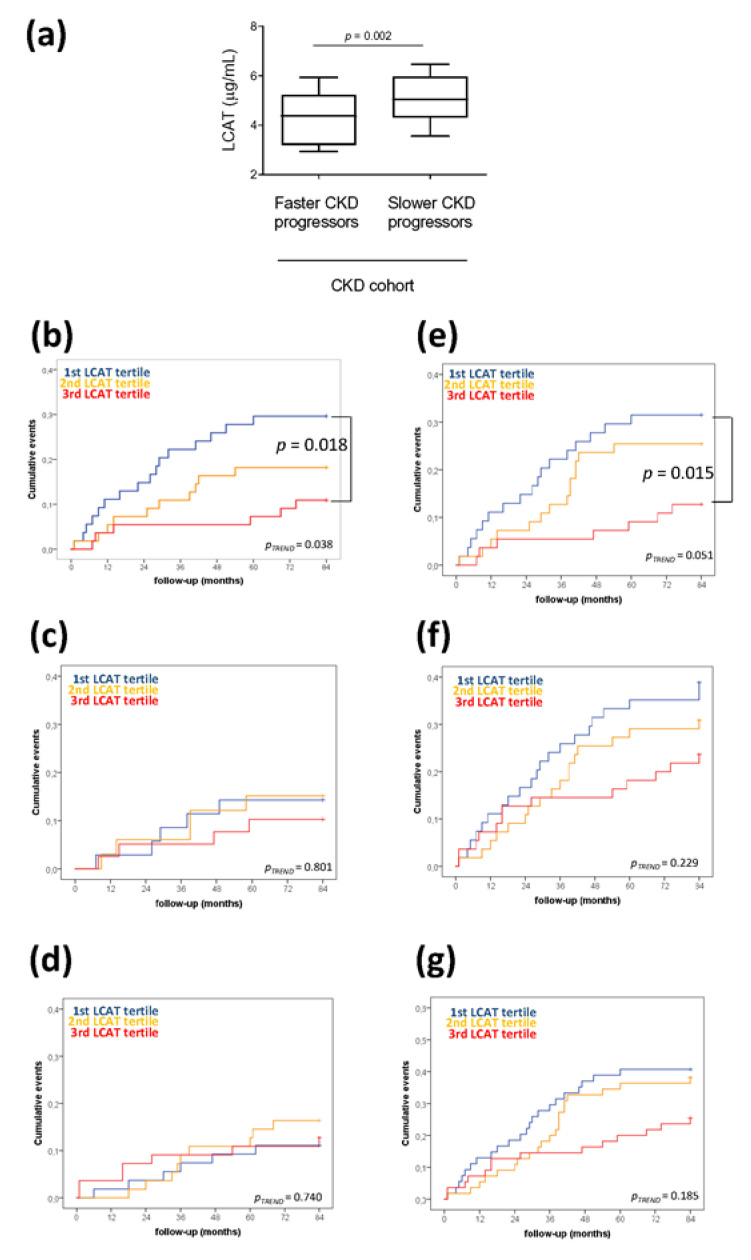
Low plasma lecithin:cholesterol acyltransferase (LCAT) concentration predicts fast chronic kidney disease (CKD) progression over time. (**a**) Differences in plasma LCAT concentration in CKD patients divided according to fast or slow CKD progression. CKD progression was defined as entry in dialysis and/or doubling of creatinine levels. *p* = 0.002 is derived from the non-parametric comparison test; data are presented as box-plots reporting median and 10th–90th percentile around the median. (**b**–**g**) Kaplan–Meier survival analyses showing an cumulative event rate in the CKD cohort divided by LCAT tertiles (1st low; 3rd high) for (**b**) dialysis entry and/or creatinine plasma levels doubling, (**c**) all-cause mortality, (**d**) cardiovascular fatal or non-fatal events, (**e**) composite outcome of dialysis entry and/or creatinine doubling and all-cause mortality, (**f**) composite outcome of dialysis entry and/or creatinine doubling and cardiovascular fatal or non-fatal events, (**g**) composite outcome of dialysis entry and/or creatinine doubling, cardiovascular fatal or non-fatal events, all-cause mortality. *p*-values are derived from log-rank test.

Among the 164 patients of the NefroPLIC cohort, 32 were identified as “fast CKD progression” patients (13 doubled basal creatinine and 19 entered dialysis during 84 months of follow-up). At the basal evaluation, these patients showed a metabolic profile similar to CKD patients who did not double basal creatinine and/or entered dialysis during follow-up (“Slow CKD progression”) (Table 1). Low HDL-c was confirmed as the unique lipid parameter reduced in patients experiencing faster CKD progression vs. those with slower progression (43.0 (14.72) vs 52.3 (14.8), *p* < 0.001, Table 1). Interestingly, also the plasma LCAT concentration was significantly reduced in patients experiencing faster CKD progression than in the slower progression group (Figure 1a) and, more importantly, was not correlated with HDL-c. Since we observed a poor statistical collinearity between the HDL-c and the LCAT concentration (Variation Inflation Factor (VIF) = 1.772 in a regression model setting LCAT as the independent variable and HDL-c as the dependent variable; VIF = 1.179 when setting HDL-c as the independent variable and LCAT as the dependent variable), we were thus prompted to investigate which was the most predictive parameter for faster CKD progression.

Cox regression model adjusted by age, gender, body mass index (BMI), systolic/diastolic blood pressure, anti-hypertensive treatments, glucose, glucose lowering treatments, albuminuria, total cholesterol, triglycerides, and lipid lowering treatments showed that reduced HDL-c is predictive of faster CKD progression (HR = 1.078 (1.016–1.143), *p* = 0.013). However, when also the LCAT concentration was included in the model, the reduction of HDL-c was of borderline statistical significance in predicting faster CKD progression (HR = 1.062 (0.999–1.129), *p* = 0.055) (Table S1). Vice versa, 1 μg/mL reduction in LCAT concentration predicted by 0.78 with up to a 3.39 times higher risk of dialysis entry and/or creatinine doubling, even when HDL-c was included in the model among other co-variates (HR = 2.370 (1.278–4.396), *p* = 0.006) (Table S2), suggesting that a reduced LCAT concentration independently predicts faster CKD progression beyond the contribution of low HDL-c.

It is worth noting that reduced LCAT concentration specifically predicted faster CKD progression and worse disease-related prognosis, as a 1 μg/mL reduction in LCAT concentration was predictive of an increased risk of a composite outcome of all-cause mortality and faster CKD progression (HR = 1.742 (1.060–2.864), *p* = 0.029), while it was not predictive for all-cause mortality alone (HR = 1.058 (0.487–2.297), *p* = 0.887) or for the occurrence of cardiovascular fatal or non-fatal events alone (HR = 0.622 (0.366–1.057), *p* = 0.079) (Table S2).

In addition, to verify whether the predictive value of the reduced LCAT concentration was also independent of other metabolic and disease-related risk factors, we divided the cohort into tertiles of LCAT concentration, and no statistical differences in all the tested parameters was observed (Table 2). Of note, patients with the lowest LCAT concentration (1st tertile) at the basal evaluation showed the highest event rate of dialysis entry and/or creatinine doubling during follow-up (Figure 1b), composite outcome of dialysis entry and/or creatinine doubling, and all-cause mortality (Figure 1e).

### 3.2. Lower Plasma LCAT Concentration Predicts Impairment of Kidney Function in General Population

To investigate if the LCAT concentration could predict the impairment of kidney function in the general population, we selected a subset of subjects with basal eGFR over 60 mL/min/1.73 m^2^ among the PLIC study cohort which were matched by age and gender with the NefroPLIC cohort. Baseline median filtration rate of the PLIC cohort was 67.51 (63.15–73.27) mL/min/1.73 m^2^, the cohort was followed for up to 84 months.

In this subset of the PLIC cohort, 1 μg/mL reduced LCAT concentration was predictive for 0.765 (0.325–1.205) mL/min reduction from basal eGFR (*p* = 0.001), when the linear regression model included age, gender, BMI, systolic and diastolic blood pressure, anti-hypertensive treatments, total cholesterol, triglycerides, HDL-c, lipid lowering drugs, glucose, oral glucose lowering drugs as co-variates (Table S3a). Vice versa, per 1 mg/dL reduction of HDL-c a mean 0.005 (−0.043–0.033) reduction in eGFR was predicted (*p* = 0.787) (Table S3b).

Of note, also in this subset of the PLIC cohort, HDL-c did not correlate with the LCAT concentration; when we divided this group into tertiles according to plasma LCAT concentration, HDL-c were 54.5 (48.0–67.2) in the 1st (low LCAT) vs. 55.0 (48.2–61.7) in 2nd vs. 59.0 (51.0–68.0) mg/dL in the third (high LCAT), *p* = 0.209). Vice versa, subjects in the lowest (1st) LCAT tertile showed higher plasma triglycerides vs. the others (96.5 (73.2–131.0 mg/dL vs. 98.0 (76.0–133.2) mg/dL of the second and vs. 84.0 (61.0–106.0) mg/dL of the third tertile, *p* = 0.039), they were taking more anti-hypertensive (35.2% vs. 32.14% the 2nd vs. 14.5% the third, *p* = 0.025) and oral glucose lowering treatments (16.7% vs. 3.6% vs. 1.8%, *p* = 0.004) (Table 3). Thus, to verify whether reduced LCAT concentration is predictive for faster eGFR reduction beyond these patterns of metabolic alterations, we also divided the subset of the PLIC cohort in LCAT tertiles (Table 3). When the annual eGFR reduction was plotted according to LCAT tertiles at baseline, subjects in the 1st tertile (mean plasma LCAT concentration of 3.89 μg/mL) showed a significantly faster progression of kidney impairment compared to subjects in the 3rd tertile (mean plasma LCAT concentration of 6.01 μg/mL) with a mean annual decline of eGFR of 2.096 (1.283–0.693) mL/min/year vs.0.693 (0.074–1.459) mL/min/year, even after adjusting for age, gender, systolic/diastolic blood pressure, anti-hypertensive treatments, glucose, oral glucose lowering therapies, total cholesterol, triglycerides, HDL-c and lipid lowering treatments (Table 4).

### 3.3. Serum from Subjects with Low Plasma LCAT Concentration Mediates ROS Production in Renal Cells and rhLCAT Limits Serum Pro-Oxidative Effect

**Figure 2:**
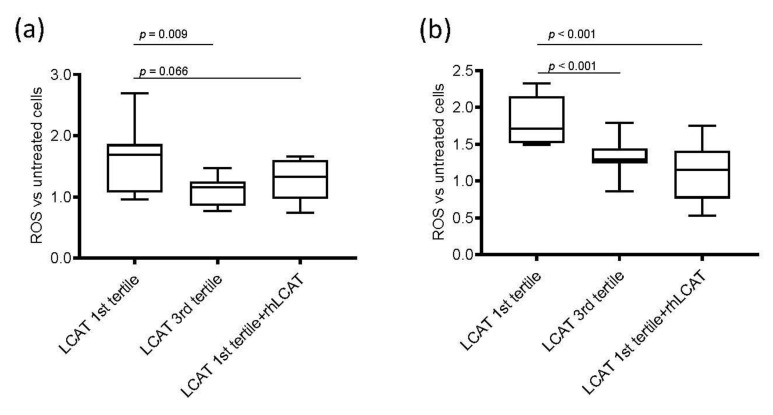
ROS production in renal cells. Box-plots showing the difference of reactive oxygen species (ROS) production after incubation with serum from subjects from the 1st and 3rd lecithin:cholesterol acyltransferase (LCAT) tertiles and with serum from subjects in 1st LCAT tertile incubated with recombinant human LCAT (rhLCAT) in (**a**) podocytes and in (**b**) tubular cells. Data are presented as box-plots reporting median and 10th–90th percentile around the median. *p*-values are derived from the Kruskall–Wallis test for comparison between each group. ROS are represented as fold versus untreated cells; the detected fluorescence was corrected for total protein and expressed as fold versus fluorescence corrected for protein content in cells not incubated with serum.

To test the hypothesis whether low plasma LCAT concentration can contribute to podocyte damage before the occurrence of changes of renal function, serum-mediated ROS production was tested in cultured podocytes and tubular cells. Serum was collected from a representative group of subjects from the subset of the PLIC cohort belonging to the 1st LCAT tertile (*n* = 11) and compared with serum obtained from subjects belonging to the 3rd LCAT tertile (*n* = 11) (Table S4). Serum of subjects in 1st LCAT tertile promoted a significant increase in ROS production in podocytes compared to that observed with serum from subjects in the 3rd LCAT tertile (Figure 2a); this effect was independent of HDL-c levels (Spearman correlation *r* = −0.125, *p* = 0.615). A similar effect was observed when tubular cells were incubated with serum from subjects in 1st LCAT tertile or in 3rd LCAT tertile (Figure 2b). In addition, podocytes incubated with serum from subjects in 1st LCAT tertile had markedly reduced podocin expression compared to podocytes incubated with serum from subjects in the 3rd LCAT tertile (1st LCAT tertile 0.78 ± 0.19 vs. 3rd LCAT tertile 1.74 ± 0.56, *p* = 0.036), suggesting a reduction in podocyte functionality under low LCAT concentration conditions.

To prove that low LCAT concentration was responsible for the increased damaging effect of serum in renal cells, serum from subjects in the 1st LCAT tertile was incubated with rhLCAT [24] to restore a concentration comparable to that of subjects in the 3rd tertile, thus allowing a proper HDL remodeling. Then, podocytes and tubular cells were incubated under these conditions (Figure 2). Interestingly, the addition of rhLCAT to sera from subjects in the 1st tertile reduced serum-mediated ROS production to a level similar to that observed with sera from 3rd LCAT tertile subjects in podocytes (Figure 2a) and, even more strikingly, in tubular cells (Figure 2b). The addition of serum-free rhLCAT to podocytes had a neutral effect on ROS production, thus suggesting that LCAT-induced modifications in lipoproteins, and specifically in HDL, may be responsible for the observed effect. Indeed, incubation of plasma with rhLCAT resulted, as expected, in a significant decrease in unesterified cholesterol and in a significant 50% reduction in the preβ-HDL particle content (Table S5).

## 4. Discussion

The main finding of our study is that the reduced plasma LCAT concentration predicts CKD progression over time in patients with renal dysfunction, and, even more striking, it predicts the impairment of kidney function in the general population. This observation extends previous cross-sectional reports by us and others showing that low HDL-c levels are typically observed in CKD patients [6,8], and that reduced LCAT concentration is a major determinant of the low HDL phenotype in CKD [7].

LCAT is a key player in lipoprotein metabolism, being the only enzyme able to esterify cholesterol in plasma and other biological fluids. LCAT is predominantly synthesized by the liver and it circulates in plasma bound to HDL, and, to a lesser extent, to apoB-containing lipoproteins. LCAT plays a major role in HDL maturation and remodeling by esterifying the free cholesterol present in nascent discoidal HDL particles [25]. Subjects with genetic LCAT deficiency present with largely immature and discoidal HDL [26] and subjects with genetic LCAT deficiency accumulate in plasma largely immature and discoidal HDL [26], large discoidal LDL particles, and the abnormal lipoprotein X [18]. The major clinical complication observed in LCAT deficient subjects is kidney dysfunction; carriers often present with proteinuria very early in life [27], and they usually develop renal failure with symptomatic edema and hypertension during the third-fourth decade of life [18]. Plasma lipoprotein alterations have been related to renal disease [17]; and, indeed, renal disease can reoccur in LCAT deficient cases who underwent kidney transplantation [28], thus supporting the systemic cause of the renal damage.

We have previously shown that CKD patients have a partial secondary LCAT deficiency [7], likely due to a down-regulation of hepatic LCAT gene expression [29], which associates with abnormalities of the HDL phenotype similar to that observed in genetic LCAT deficiency. These abnormalities include low plasma HDL-c and apoA-I levels, a low level of particles containing apoA-I and apoA-II, and a high content of discoidal preβ-HDL [7]. This peculiar profile is clearly related to the residual amount of circulating LCAT, as shown by the gene-dose dependent effect observed for these biomarkers in carriers of genetic LCAT deficiency [30].

Of note, in our CKD patients, low HDL-c levels and reduced LCAT concentration independently predicted faster CKD progression. HDL-c and LCAT do not correlate in the present study, differently from what was observed in end stage renal disease patients [7] but in agreement with the absence of correlation observed in the general population [31]. The predictive value of the reduced LCAT concentration was unaffected by the presence of albuminuria, and when apoA-I was included in the model (Table S6), a finding coupled with lack of correlation between LCAT and basal eGFR, blood urea nitrogen, albuminuria, or urinary-to-creatinine ratio. Besides, both low HDL-c and LCAT concentration predicted disease progression in diabetic CKD patients (97 out of 164 from the nefroPLIC cohort) and renal function worsening in non-diabetic subjects (153 out of 165 from the PLIC cohort) (Table S7).

Taken together, the present findings highlight that the compromised plasma cholesterol esterification is relevant for CKD progression and renal function impairment even at initial stages of renal dysfunction. At the same time, these data call for the involvement of LCAT in the development of renal damage. We have shown that sera from subjects with low LCAT concentration, characterized by an increased unesterified/total cholesterol ratio and accumulation of discoidal preβ-HDL (Table S4), present with pro-oxidative effects on podocytes and tubular cells and impair podocin expression that may contribute to the onset and progression of renal dysfunction observed later in these subjects [32]. The causal relation between low LCAT concentration and increased serum-mediated ROS production was confirmed by the demonstration that in vitro addition to sera in the low LCAT group of a physiological amount of rhLCAT can correct HDL abnormalities associated with low LCAT levels, and it reduces serum-mediated ROS production in renal cells. Most importantly, LCAT protective and anti-oxidant functions are not exerted directly by the enzyme, but result from the modifications induced by the enzyme in plasma lipoproteins, and specifically on HDL particles. Transient LCAT overexpression was already shown to be associated with a reduction of oxidative stress in a mouse model characterized by impaired HDL antioxidant capacity [33].

In conclusion, this is the first study that prospectively addresses the impact of plasma LCAT levels in predicting renal disease onset and progression. Our data demonstrate that reduced circulating LCAT levels predict CKD progression at early stages of renal dysfunction independent of changes in HDL-c levels, supporting the hypothesis that changes in HDL subclass distribution, besides HDL-c levels [6] contribute to the progression of renal damage. Our in vitro data support the hypothesis that the pharmacological modulation of LCAT, either with the recombinant protein or with small molecule activators, can improve the abnormal HDL phenotype in CKD, thus reducing serum pro-oxidative effects associated with low LCAT. Whether LCAT modulation could also restore the defective functionality of CKD HDL [12,34,35], thus contributing to an amelioration of the cardiovascular profile of CKD patients, remains to be determined and should be tested in future studies.

## Figures and Tables

**Table 1:** Clinical and biochemical characteristics of the CKD cohort.

	Entire CKD Cohort	Slow CKD Progression	Fast CKD Progression	*p*
*N*	164	132	32	
Age (years)	68 (61–75)	68 (59–74)	73 (66–79)	<0.001
Gender (men, *n* (%))	107 (65.2)	86 (65.1)	21 (65.6)	0.957
BMI (kg/m^2^)	29.44 (5.46)	29.64 (5.55)	28.67 (5.24)	0.589
Waist/hip	0.970 (0.071)	0.969 (0.069)	0.974 (0.083)	0.568
Systolic Blood Pressure (mmHg)	144 (18)	144 (18)	144 (21)	0.418
Diastolic Blood Pressure (mmHg)	83 (12)	83 (10)	84 (18)	0.283
ACE Inhibitors (*n* (%), yes)	77 (46.9)	63 (47.7)	14 (43.7)	0.976
ARBs (*n* (%), yes)	54 (32.9)	43 (32.6)	11 (34.4)	0.609
ACE Inhibitors + ARBs Association (*n* (%), yes)	31 (18.9)	23 (17.4)	8 (25.0)	0.326
Diuretics (*n* (%), yes)	59 (35.9)	46 (34.8)	13 (40.6)	0.383
Diuretics + ARBs Association (*n* (%), yes)	28 (17.1)	21 (15.9)	7 (21.8)	0.421
Beta-Blockers (*n* (%), yes)	30 (18.3)	28 (21.2)	2 (6.2)	0.064
C-Reactive Protein (mg/L)	0.23 (0.12–0.45)	0.37 (0.12–0.41)	0.43 (0.18–0.51)	0.149
Fasting Glucose Levels (mg/dL)	126.5 (100.0–157.5)	132.0 (103.0–165.0)	112.5 (94.0–147.0)	0.044
Glycated Hemoglobin (%)	6.6 (6.1–7.4)	6.8 (6.2–7.4)	6.2 (6.0–6.9)	0.104
OGLT (*n* (%), yes)	70 (42.7)	60 (45.4)	10 (31.2)	0.145
OGLT Vintage Before Basal Evaluation (months)	84 (36–180)	84 (36–180)	120 (31–168)	0.952
Insulin Analogues (*n* (%), yes)	27 (16.4)	23 (17.4)	4 (12.5)	0.500
Insulin Analogues Vintage Before Basal Evaluation (months)	24 (12–120)	24 (12–120)	48 (7–108)	0.846
Creatinine (mg/dL)	1.52 (1.00–2.02)	1.30 (0.90–1.80)	2.77 (1.8–4.1)	<0.001
eGFR (mL/min/1.73 m^2^)	44.65 (30.00–68.46)	49.42 (35.06–71.23)	19.55 (16.01–35.63)	<0.01
Urinary Creatinine/Albumin (mg/g)	6.68 (1.74–36.14)	5.55 (1.56–26.61)	39.93 (2.74–276.73)	0.004
Urinary Albumin (mg/L)	33.66 (10.30–228.33)	28.06 (9.85–174.85)	434.53 (47.80–1049.55)	0.001
Blood Urea Nitrogen (mg/dL)	52.00 (40.00–82.00)	49.85 (38.02–69.52)	98.50 (61.67–127.77)	<0.001
Calcium (mmol/L)	2.36 (2.30–2.44)	2.36 (2.30–2.44)	2.35 (2.24–2.43)	0.543
Phosphorous (mmol/L)	1.09 (0.94–1.20)	1.05 (0.94–1.18)	1.21 (1.05–1.33)	0.005
Total Cholesterol (mg/dL)	201.5 (46.2)	197.2 (43.4)	217.8 (54.8)	0.133
Triglycerides (mg/dL)	136.0 (88.0–187.0)	128.0 (87.2–182.0)	143.5 (101.7–223.0)	0.061
LDL-Cholesterol (mg/dL)	120.9 (42.0)	116.6 (38.7)	136.3 (51.2)	0.086
HDL-Cholesterol (mg/dL)	50.40 (15.22)	51.94 (14.60)	44.40 (15.07)	0.010
Non-HDL-Cholesterol (mg/dL)	151.1 (46.8)	145.2 (43.5)	173.4 (53.9)	0.013
Apolipoprotein A-I (mg/dL)	147.0 (131.0–164.0)	148.0 (132.0–166.0)	143.5 (121.5–152.7)	0.043
Apolipoprotein B (mg/dL)	127.0 (111.0–146.0)	126.0 (111.0–142.0)	130.5 (114.2–169.5)	0.385
Hypolipidemic Treatments, Statins (*n*, yes)	57 (34.5)	50 (37.8)	7 (21.8)	0.152
Hypolipidemic Treatments, Fibrates (*n*, yes)	7 (4.2)	5 (3.7)	2 (6.2)	0.484
Anti-Platelets (ASA) (*n*, yes)	64 (39.0)	54 (40.9)	10 (31.2)	0.444

Data are reported as mean (SD) or median (Interquartile Range, as the range between the 25th and the 75th percentile around each median value). *p*-values indicate differences between the two chronic kidney disease (CKD) groups. “ARBs”: angiotensin receptor blockers; ‘’ASA’’: acetilsalycilic acid or other anti-platelets; “OLGT”: Oral glucose lowering treatments; ‘’BMI’’: body mass index; ‘’ACE’’: angiotensin-converting enzyme; ‘’eGFR’’: estimated glomerular filtration rate; ‘’LDL’’: low density lipoprotein; ‘’HDL’’: high-density lipoprotein.

**Table 2:** Clinical and biochemical characteristics of the CKD cohort per LCAT tertiles.

	LCAT Tertiles			
LCAT (μg/mL) (Mean (Range))	1st (*n* = 54) 3.65 (3.16–3.95)	2nd (*n* = 55) 4.91 (4.60–5.18)	3rd (*n* = 55) 6.13 (5.80–647)	*_p_*_TREND_
Age (years)	68 (62–71)	70 (64–75)	66 (58–75)	0.232
Gender (men, *n* (%))	39 (72.2)	34 (61.3)	34 (61.3)	0.421
BMI (kg/m^2^)	29.22 (25.68–33.36)	27.91 (24.87–32.23)	27.88 (25.71–32.38)	0.480
Waist/hip	0.97 (0.07)	0.95 (0.07)	0.98 (0.07)	0.275
Systolic Blood Pressure (mmHg)	145 (131–160)	140 (130–155)	140 (130–160)	0.620
Diastolic Blood Pressure (mmHg)	80 (80–90)	80 (80–90)	85 (80–90)	0.266
ACE Inhibitors (*n* (%), yes)	24 (44.4)	26 (47.3)	27 (49.1)	0.973
ARBs (*n* (%), yes)	14 (25.9)	19 (34.5)	21 (38.2)	0.495
ACE Inhibitors + ARBs Association (*n* (%), yes)	8 (14.8)	10 (18.1)	13 (23.6)	0.518
Diuretics (*n* (%), yes)	18 (33.3)	22 (40)	19 (34.5)	0.815
Diuretics + ARBs Association (*n* (%), yes)	7 (13.0)	10 (18.1)	11 (20.0)	0.627
Beta-Blockers (*n* (%), yes)	6 (11.1)	12 (21.8)	12 (21.8)	0.313
C-Reactive Protein (mg/L)	0.26 (0.15–0.50)	0.28 (0.10–0.49)	0.19 (0.11–0.40)	0.341
Fasting Glucose Levels (mg/dL)	135.5 (101.0–174.5)	123.0 (102.0–159.0)	123.0 (99.0–154.0)	0.777
Glycated Hemoglobin (%)	6.55 (6.10–7.47)	6.60 (6.00–7.40)	7.00 (6.30–7.55)	0.324
OGLT (*n* (%), yes)	20 (37.1)	26 (47.2)	24 (43.6)	0.549
OGLT Vintage (Months Before Basal Evaluation)	72 (36–174)	78 (36–210)	90 (27–180)	0.666
Insulin Analogues (*n* (%), yes)	6 (11.1)	14 (25.4)	7 (12.7)	0.086
Insulin Analogues Vintage (Months Before Basal Evaluation)	60 (9–120)	24 (12–120)	48 (11–156)	0.849
Creatinine (mg/dL)	1.63 (1.09–2.13)	1.16 (0.90–1.90)	1.61 (1.10–1.85)	0.238
eGFR (mL/min/1.73 m^2^)	47.56 (25.43–48.91)	44.49 (32.82–73.67)	42.24 (29.73–59.36)	0.738
Urinary Creatinine/Albumin (mg/g)	6.14 (2.24–73.90)	8.57 (1.62–28.91)	5.45 (1.21–37.11)	0.663
Urinary Albumin (mg/L)	30.80 (12.50–316.20)	39.43 (10.27–203.00)	32.90 (9.44–225.63)	0.890
Blood Urea Nitrogen (mg/dL)	52.0 (40.5–85.7)	50.9 (37.1–70.8)	56.5 (44.9–86.3)	0.477
Calcium (mmol/L)	2.37 (2.33–2.44)	2.34 (2.27–2.44)	2.37 (2.30–2.43)	0.401
Phosphorous (mmol/L)	1.12 (0.18)	1.05 (0.18)	1.06 (0.18)	0.178
Total Cholesterol (mg/dL)	204.6 (50.3)	200.6 (41.2)	198.4 (47.4)	0.512
Triglycerides (mg/dL)	140.0 (93.2–189.2)	122.0 (88.0–183.0)	143.0 (88.0–176.0)	0.723
LDL-Cholesterol (mg/dL)	123.1 (45.7)	120.7 (36.1)	117.4 (44.3)	0.487
HDL-Cholesterol (mg/dL)	46.0 (37.0–61.0)	49.0 (42.0–60.0)	48.0 (42.0–57.0)	0.469
Non-HDL-cholesterol (mg/dL)	161.0 (120.7–185.5)	147.0 (119.0–175.0)	142.0 (119.0–167.0)	0.436
Apolipoprotein A-I (mg/dL)	146.8 (32.1)	148.8 (27.4)	151.9 (29.7)	0.893
Apolipoprotein B (mg/dL)	130.6 (33.1)	129.8 (28.6)	132.02 (32.5)	0.999
Hypolipidemic Treatments, Statins (*n*, yes)	19 (35.2)	14 (25.4)	24 (43.6)	0.132
Hypolipidemic Treatments, Fibrates (*n*, yes)	1 (1.8)	5 (9.1)	1 (1.8)	0.104
Anti-Platelets (ASA) (*n*, yes)	23 (42.6)	22 (40.0)	19 (34.5)	0.547

Data are reported as mean (SD) or median (interquartile range, as the range between the 25th and the 75th percentile around each median value). “ARBs”: angiotensin receptor blockers; ‘’ASA“: acetilsalycilic acid or other anti-platelets; “OLGT”: oral glucose lowering treatments; ’’BMI’’: body mass index; ‘’ACE’’: angiotensin-converting enzyme; ‘’eGFR’’: estimated glomerular filtration rate; ‘’LDL’’: low-density lipoprotein; ‘’HDL’’: high-density lipoprotein; ‘’CKD’’: chronic kidney disease; ‘’LCAT’’: lecithin:cholesterol acyltransferase.

**Table 3:** Clinical and biochemical characteristics of the PLIC cohort at basal visit per LCAT tertiles.

LCAT (μg/mL) (Mean (Range))	Entire Cohort		LCAT Tertiles		
	(*n* = 165) 4.85 (4.22–5.58)	1st (*n* = 54) 3.89 (3.34–4.21)	2nd (*n* = 56) 4.82 (4.67–5.09)	3rd (*n* = 55) 6.01 (5.00–6.34)	*p*_TREND_
Age (years)	68 (66–72)	69 (66–73)	67 (65–72)	69 (66–72)	0.742
Gender (men, *n* (%))		26 (48.1)	27 (48.2)	27 (49.1)	0.994
BMI (kg/m^2^)	26.31 (24.53–28.70)	26.49 (24.37–29.15)	25.85 (24.16–29.25)	26.33 (24.86–28.07)	0.949
Waist/hip	0.89 (0.07)	0.89 (0.07)	0.90 (0.08)	0.89 (0.07)	0.826
Systolic Blood Pressure (mmHg)	135 (120–150)	135 (120–149)	132 (120–150)	140 (120–150)	0.860
Diastolic Blood Pressure (mmHg)	80 (75–80)	80 (76–80)	80 (75–80)	80 (70–80)	0.431
Anti-Hypertensive Treatments (*n* (%), yes)	45 (27.3)	19 (35.2)	18 (32.14)	8 (14.5)	0.025
C-Reactive Protein (mg/L)	0.19 (0.08–0.39)	0.16 (0.04–0.32)	0.19 (0.09–0.38)	0.20 (0.07–0.41)	0.592
Fasting Glucose Levels (mg/dL)	98.0 (90.0–107.0)	99.0 (90.0–110.2)	96.0 (91.0–104.0)	97.0 (90.0–109.0)	0.614
OGLT (*n* (%), yes)	12 (7.3)	9 (16.7)	2 (3.6)	1 (1.8)	0.004
OGLT Vintage (Months Before Basal Evaluation)	32(24–80)	24 (22–56)	80; 80 ∗	80	0.051
Creatinine (mg/dL)	0.91 (0.80–1.03)	0.92 (0.79–1.00)	0.89 (0.79–1.02)	0.96 (0.80–1.08)	0.418
eGFR (mL/min/1.73 m^2^)	67.51 (63.15–73.27)	68.71 (63.78–74.67)	68.14 (63.74–74.36)	66.52 (62.02–72.05)	0.178
Total Cholesterol (mg/dL)	232.8 (44.94)	223.4 (48.7)	237.2 (45.9)	237.8 (38.8)	0.112
Triglycerides (mg/dL)	95.5 (69.2–123.7)	96.5 (73.2–131.0)	98.0 (76.0–133.2)	84.0 (61.0–106.0) *^,†^	0.039
LDL-Cholesterol (mg/dL)	153.0 (41.4)	142.0 (42.9)	157.5 (43.0)	158.9 (36.1)	0.080
HDL-Cholesterol (mg/dL)	56.0 (49.0–65.7)	54.5 (48.0–67.2)	55.0 (48.2–61.7)	59.0 (51.0–68.0)	0.209
Non-HDL-Cholesterol (mg/dL)	173.0 (142.2–206.0)	166.0 (134.5–189.2)	180.0 (144.0–213.0)	176.0 (146.0–204.0)	0.133
Apolipoprotein A-I (mg/dL)	153.3 (22.7)	152.3 (21.9)	151.0 (23.9)	157.7 (21.1)	0.160
Apolipoprotein B (mg/dL)	123.1 (35.36)	115.4 (37.3)	126.6 (36.9)	127.4 (31.0)	0.111
Hypolipidemic Treatments, Statins (*n*, yes)	53 (32.2)	15 (27.8)	19 (33.9)	19 (34.5)	0.717
Anti-Platelets (ASA) (*n*, yes)	34 (20.6)	12 (22.2)	12 (21.4)	10 (18.2)	0.937

Data are reported as mean (SD) or median (Interquartile Range, as the range between the 25th and the 75th percentile around each median value). * *p* < 0.05 vs. 1st LCAT tertile; ^†^ *p* < 0.05 vs. 2nd LCAT tertile; ∗ raw data for *n* = 2 subjects are reported. ‘’ASA “: Acetilsalycilic Acid or other anti-platelets; ‘’OLGT”: oral glucose lowering treatments; ’’BMI’’: body mass index; ‘’ACE’’: angiotensin-converting enzyme; ‘’eGFR’’: estimated glomerular filtration rate; ‘’LDL’’: low-density lipoprotein; ‘’HDL’’: high-density lipoprotein; ‘’LCAT’’: lecithin:cholesterol acyltransferase.

**Table 4:** Annual eGFR reduction per LCAT tertiles.

Model	1st Tertile	2nd Tertile	3rd Tertile
1	−2.249 (−1.524– −2.974)	−1.273 (−0.562– −1.985)	−0.533 (−0.186– −1.251) (*p* = 0.018 vs. 1st tertile)
2	−2.260 (−1.550– −2.969)	−1.279 (−0.582– −1.976)	−0.516 (−0.187– −1.220) (*p* = 0.014 vs. 1st tertile)
3	−2.015 (−1.278– −2.752)	−1.344 (−0.645– −2.042)	−0.684 (−0.043– −1.411) (*p* = 0.001 vs. 1st tertile)
4	−2.096 (−1.283– −2.908)	−1.260 (−0.522– −1.998)	−0.693 (−0.074– −1.459) *p* = 0.001 vs. 1st tertile

Data are reported as mean (lower and upper bound around the mean value) of annual estimated glomerular filtration rate (eGFR) reduction. *p-*values are derived from ANCOVA (Bonferroni post-hoc) with Model 1: unadjusted; Model 2: adjusting for age and gender; Model 3: adjusting for age, gender, body mass index (BMI), systolic and diastolic blood pressure, glucose, total cholesterol, triglycerides, high-density lipoprotein-cholesterol (HDL-c); Model 4: adjusting for age, gender, BMI, systolic and diastolic blood pressure, anti-hypertensive treatments, glucose, oral glucose lowering treatments, total cholesterol, triglycerides, HDL-c, hypolipideamic treatments. ‘’LCAT’’: lecithin:cholesterol acyltransferase.

## Supplementary Materials

The following are available online at https://www.mdpi.com/2077-0383/9/7/2289/s1. Table S1: Reduced HDL-c predicts faster CKD progression, but dependently from reduced LCAT concentration, Table S2: Reduced LCAT concentration specifically predicts risk of dialysis entry and/or creatinine doubling and all-cause mortality, Table S3: Reduced LCAT concentration predicts higer annual eGFR reduction more significantly than reduced HDL-c, Table S4: Characteristics of subjects enrolled in the in vitro studies, Table S5: Lipid levels after incubation of plasma with or without rhLCAT, Table S6: Reduced LCAT concentration predicts faster CKD progression independently from ApoA-I circulating levels, Table S7: Predictive value of reduced HDL-c and reduced LCAT concentration for CKD progression and renal function impairment in patients with and without diabetes.
