# Supplementary material for: Low Plasma Lecithin: Cholesterol Acyltransferase (LCAT) Concentration Predicts Chronic Kidney Disease
Source: J Clin Med. 2020 Jul 18;9(7):2289. doi: 10.3390/jcm9072289 (PMC7408930; doi:10.3390/jcm9072289)
Supplement: Supplementary file 1 [file jcm-09-02289-s001.docx]

Supplementary Materials

**Table S1.** Reduced HDL-c predicts faster CKD progression but dependently from reduced LCAT concentration.

|  |  |  |  |  |  | **95% C.I.** | |  |
| --- | --- | --- | --- | --- | --- | --- | --- | --- |
|  | **model** | **beta** | **S.E.** | **Wald F** | **HR** | **Lower** | **Upper** | ***P*** |
| Per 1 mg/dL in HDL-c reduction | 1 | 0.053 | 0.015 | 12.02 | 1.054 | 1.023 | 1.086 | 0.001 |
|  | 2 | 0.062 | 0.016 | 14.89 | 1.064 | 1.031 | 1.098 | <0.001 |
|  | 3 | 0.079 | 0.031 | 6.63 | 1.082 | 1.019 | 1.148 | 0.016 |
|  | 4 | 0.079 | 0.031 | 6.22 | 1.078 | 1.016 | 1.143 | 0.013 |
|  | 5 | 0.060 | 0.031 | 3.676 | 1.062 | 0.999 | 1.129 | 0.055 |

Table reports Hazard Risks (HR) (with lower and upper bounds of the 95% Confidence Interval (C.I.)) for dialysis entry and/or creatinine doubling in the nefroPLIC cohort per one mg/dL reduction in HDL-c.S.E.: standard error of the beta coefficient; Wald F: from Wald statistic for forward selection of co-variates (with entry testing (set at 0.05) and removal testing (set at 0.1)); *p*-value. Cox regression models were adjusted as follows: Model 1 is un-adjusted; Model 2 is adjusted by age and gender; Model 3 is adjusted by age, gender, BMI, systolic and diastolic blood pressure, albuminuria, total cholesterol, triglycerides and glucose; Model 4 is adjusted by age, gender, BMI, systolic and diastolic blood pressure, anti-hypertensive treatments, albuminuria, total cholesterol, triglycerides, lipid lowering treatments, glucose, oral glucose lowering treatments; Model 5 is adjusted by age, gender, BMI, systolic and diastolic blood pressure, anti-hypertensive treatments, albuminuria, total cholesterol, triglycerides, LCAT concentration, lipid lowering treatments, glucose, oral glucose lowering treatments.

**Table S2.** Reduced LCAT concentration specifically predicts risk of dialysis entry and/or creatinine doubling and all-cause mortality.

|  |  |  |  |  |  | **95% C.I.** | |  |
| --- | --- | --- | --- | --- | --- | --- | --- | --- |
|  | **model** | **beta** | **S.E.** | **Wald F** | **HR** | **Lower** | **Upper** | **P** |
| Dialysis entry and/or creatinine doubling | | | | | | | | |
| Per 1 μg/mL reduction in LCAT concentration | 1 | 0.518 | 0.161 | 10.35 | 1.679 | 1.224 | 2.302 | 0.001 |
|  | 2 | 0.554 | 0.167 | 11.03 | 1.740 | 1.255 | 2.412 | 0.001 |
|  | 3 | 0.679 | 0.266 | 6.52 | 1.972 | 1.171 | 3.322 | 0.011 |
|  | 4 | 0.854 | 0.311 | 7.55 | 2.348 | 1.278 | 4.317 | 0.006 |
|  | 5 | 0.863 | 0.315 | 7.49 | 2.370 | 1.278 | 4.396 | 0.006 |
| All-cause mortality | | | | | | | | |
| Per 1 μg/mL reduction in LCAT concentration | 1 | 0.117 | 0.197 | 0.350 | 1.124 | 0.763 | 1.655 | 0.554 |
|  | 2 | 0.103 | 0.203 | 0.258 | 1.109 | 0.744 | 1.652 | 0.612 |
|  | 3 | 0.089 | 0.355 | 0.063 | 1.093 | 0.545 | 2.191 | 0.802 |
|  | 4 | 0.061 | 0.398 | 0.024 | 1.063 | 0.488 | 2.318 | 0.878 |
|  | 5 | 0.056 | 0.396 | 0.020 | 1.058 | 0.487 | 2.297 | 0.887 |
| Cardiovascular fatal or non-fatal events | | | | | | | | |
| Per 1 μg/mL reduction in LCAT concentration | 1 | 0.082 | 0.189 | 0.187 | 0.922 | 0.637 | 1.335 | 0.666 |
|  | 2 | 0.100 | 0.191 | 0.273 | 0.905 | 0.623 | 1.315 | 0.601 |
|  | 3 | 0.446 | 0.262 | 2.911 | 0.640 | 0.383 | 1.069 | 0.088 |
|  | 4 | 0.473 | 0.270 | 3.082 | 0.623 | 0.367 | 1.057 | 0.079 |
|  | 5 | 0.476 | 0.271 | 3.086 | 0.622 | 0.366 | 1.057 | 0.079 |
| Sum of dialysis entry and/or creatinine doubling + all-cause mortality | | | | | | | | |
| Per 1 μg/mL reduction in LCAT concentration | 1 | 0.403 | 0.145 | 7.704 | 1.497 | 1.126 | 1.990 | 0.006 |
|  | 2 | 0.428 | 0.150 | 8.177 | 1.534 | 1.144 | 2.057 | 0.004 |
|  | 3 | 0.527 | 0.239 | 4.874 | 1.694 | 1.061 | 2.705 | 0.027 |
|  | 4 | 0.567 | 0.255 | 4.934 | 1.762 | 1.069 | 2.906 | 0.026 |
|  | 5 | 0.555 | 0.254 | 4.788 | 1.742 | 1.060 | 2.864 | 0.029 |
| Sum of dialysis entry and/or creatinine doubling + fatal and non-fatal cardiovascular events | | | | | | | | |
| Per 1 μg/mL reduction in LCAT concentration | 1 | 0.287 | 0.125 | 5.253 | 1.332 | 1.024 | 1.703 | 0.022 |
|  | 2 | 0.301 | 0.129 | 5.459 | 1.352 | 1.050 | 1.740 | 0.019 |
|  | 3 | 0.112 | 0.173 | 0.421 | 1.119 | 0.797 | 1.569 | 0.516 |
|  | 4 | 0.117 | 0.179 | 0.429 | 1.124 | 0.792 | 1.595 | 0.512 |
|  | 5 | 0.114 | 0.179 | 0.407 | 1.121 | 0.789 | 1.592 | 0.524 |
| Sum of dialysis entry and/or creatinine doubling + all-cause mortality + fatal and non-fatal cardiovascular events | | | | | | | | |
| Per 1 μg/mL reduction in LCAT concentration | 1 | 0.242 | 0.117 | 4.236 | 1.273 | 1.012 | 1.603 | 0.040 |
|  | 2 | 0.253 | 0.120 | 4.420 | 1.288 | 1.017 | 1.631 | 0.036 |
|  | 3 | 0.083 | 0.168 | 0.245 | 1.087 | 0.782 | 1.509 | 0.620 |
|  | 4 | 0.071 | 0.172 | 0.172 | 1.074 | 0.766 | 1.506 | 0.679 |
|  | 5 | 0.068 | 0.172 | 0.155 | 1.070 | 0.764 | 1.500 | 0.693 |

Table reports Hazard Risks (HR) (with lower and upper bounds of the 95% Confidence Interval (C.I.)) for each of the studied endpoints of the study in the nefroPLIC cohort per one one ug/mL reduction in LCAT concentration. S.E.: standard error of the beta coefficient; Wald F: from Wald statistic for forward selection of co-variates (with entry testing (set at 0.05) and removal testing (set at 0.1)); *p*-value. Model 1 is un-adjusted; Model 2 is adjusted by age and gender; Model 3 is adjusted by age, gender, BMI, systolic and diastolic blood pressure, albuminuria, total cholesterol, triglycerides and glucose; Model 4 is adjusted by age, gender, BMI, systolic and diastolic blood pressure, anti-hypertensive treatments, albuminuria, total cholesterol, triglycerides, lipid lowering treatments, glucose, oral glucose lowering treatments; Model 5 is adjusted by age, gender, BMI, systolic and diastolic blood pressure, anti-hypertensive treatments, albuminuria, total cholesterol, triglycerides, HDL-c, lipid lowering treatments, glucose, glucose lowering treatments.

**Table S3.** Reduced LCAT concentration predicts higher annual eGFR reduction more significantly than reduced HDL-c.

**(A)**

|  | **model** | **beta** | **Lower** | **Upper** | **t** | **P** |
| --- | --- | --- | --- | --- | --- | --- |
| Per 1 μg/mL in LCAT reduction | 1 | 0.852 | 0.451 | 1.253 | 4.197 | <0.001 |
|  | 2 | 0.838 | 0.445 | 1.232 | 4.207 | <0.001 |
|  | 3 | 0.727 | 0.324 | 1.130 | 3.564 | <0.001 |
|  | 4 | 0.766 | 0.328 | 1.204 | 3.455 | 0.001 |
|  | 5 | 0.765 | 0.325 | 1.205 | 3.440 | 0.001 |

**(B)**

|  | **model** | **beta** | **Lower** | **Upper** | **t** | **P** |
| --- | --- | --- | --- | --- | --- | --- |
| Per 1 mg/dL in HDL-c reduction | 1 | -0.027 | -0.058 | 0.004 | -1.720 | 0.087 |
|  | 2 | -0.030 | -0.061 | 0.002 | -1.877 | 0.062 |
|  | 3 | -0.005 | -0.042 | 0.032 | -0.266 | 0.791 |
|  | 4 | -0.006 | -0.045 | 0.033 | -0.027 | 0.771 |
|  | 5 | -0.005 | -0.043 | 0.033 | -0.270 | 0.787 |

Table reports annual reduction in eGFR per both each ug/mL reduction in LCAT concentration (A) and per one mg/dL in HDL-c reduction (B). Beta coefficients are reported, with the lower and upper bounds around beta, the t-value (the size of the difference relative to the variation in HDL-c or LCAT concentration) and the *p*-value. Model 1 is un-adjusted; Model 2 is adjusted by age and gender; Model 3 is adjusted by age, gender, BMI, systolic and diastolic blood pressure, total cholesterol, triglycerides and glucose; Model 4 is adjusted by age, gender, BMI, systolic and diastolic blood pressure, anti-hypertensive treatments, total cholesterol, triglycerides, lipid lowering treatments, glucose, oral glucose lowering treatments; Model 5 is adjusted by age, gender, BMI, systolic and diastolic blood pressure, anti-hypertensive treatments, total cholesterol, triglycerides, HDL-c, lipid lowering treatments, glucose, oral glucose lowering treatments.

**Table S4.** Characteristics of subjects enrolled in the in vitro studies.

|  | **1st LCAT tertile** | **3rd LCAT tertile** | **P** |
| --- | --- | --- | --- |
| N total | 11 | 11 |  |
| Age (years) | 68 (6) | 68 (3) | 1.000 |
| Gender (men, n,(%)) | 5 (45) | 4 (36) | 1.000 |
| BMI (Kg/m2) | 26.12 (2.86) | 27.12 (1.44) | 0.313 |
| LCAT mass (μg/ml) | 2.94 (0.44) | 6.01 (1.00) | *<0.001* |
| Creatinine (mg/dL) | 0.87 (0.12) | 0.92 (0.12) | 0.340 |
| eGFR (mL/min/1.73 m2) | 70.40 (5.74) | 68.42 (5.12) | 0.403 |
| Total cholesterol (mg/dL) | 239.6 (57.4) | 241.1 (38.6) | 0.943 |
| Unesterified cholesterol (mg/dL) | 51.3 (12.3) | 46.4 (10.1) | 0.403 |
| Unesterified/Total cholesterol | 0.21 (0.01) | 0.19 (0.03) | *0.022* |
| Triglycerides (mg/dL) | 116.7 (60.8) | 80.8 (43.7) | 0.127 |
| LDL-cholesterol (mg/dL) | 160.5 (51.6) | 161.4 (38.7) | 0.964 |
| HDL-cholesterol (mg/dL) | 55.8 (13.2) | 63.5 (12.8) | 0.180 |
| Non-HDL-cholesterol (mg/dL) | 183.8 (50.9) | 177.5 (41.2) | 0.753 |
| Apolipoprotein A-I (mg/dL) | 146.7 (22.5) | 161.1 (16.6) | 0.103 |
| Apolipoprotein B (mg/dL) | 130.3 (47.1) | 126.1 (25.5) | 0.797 |
| Pre-beta HDL (% of total apoA-I) | 22.4 (4.9) | 15.9 (5.8) | *0.015* |

Data are expressed as mean(SD).

**Table S5.** Lipid levels after incubation of plasma with or without rhLCAT.

|  | **-rhLCAT** | **+rhLCAT** | **P** |
| --- | --- | --- | --- |
| N | 11 | 11 |  |
| Total cholesterol (mg/dL) | 239.6 (57.4) | 239.4 (58.2) | 0.992 |
| Unesterified cholesterol (mg/dL) | 51.3 (12.3) | 44.1 (10.3) | 0.152 |
| Unesterified/Total cholesterol | 0.21 (0.01) | 0.19 (0.02) | *<0.001* |
| LDL-cholesterol (mg/dL) | 160.5 (51.6) | 158.8 (51.8) | 0.940 |
| HDL-cholesterol (mg/dL) | 55.8 (13.2) | 57.0 (13,5) | 0.833 |
| Non-HDL-cholesterol (mg/dL) | 183.8 (51.0) | 182.4 (51.5) | 0.948 |
| Triglycerides (mg/dL) | 116.7 (60.9) | 117.2 (60.6) | 0.985 |
| Apolipoprotein A-I (mg/dL) | 146.7 (22.5) | 147.1 (22.7) | 0.968 |
| Apolipoprotein B (mg/dL) | 130.3 (47.0) | 128.9 (46.8) | 0.948 |
| Pre-beta HDL (% of total apoA-I) | 22.4 (4.9) | 11.0 (1.4) | *0.004* |

**Table S6.** Reduced LCAT concentration predicts faster CKD progression independently from ApoA-I circulating levels.

|  |  |  |  |  | **95% C.I.** | |  |
| --- | --- | --- | --- | --- | --- | --- | --- |
|  | **beta** | **S.E.** | **Wald F** | **HR** | **Lower** | **Upper** | **P** |
| Per 1 μg/mL reduction in LCAT concentration | 0.746 | 0.269 | 7.68 | 2.108 | 1.244 | 3.571 | 0.006 |

Table reports Hazard Risks (HR) (with lower and upper bounds of the 95% Confidence Interval (C.I.)) for dialysis entry and/or creatinine doubling in the nefroPLIC cohort per one ug/mL reduction in LCAT concentration. S.E.: standard error of the beta coefficient; Wald F: from Wald statistic for forward selection of co-variates (with entry testing (set at 0.05) and removal testing (set at 0.1)); *p*-value. The model includes: age, gender, BMI, systolic and diastolic blood pressure, anti-hypertensive treatments, albuminuria, total cholesterol, triglycerides, HDL-c, ApoA-I, lipid lowering treatments, glucose, oral glucose lowering treatments.

**Table S7.** Predictive value of reduced HDL-c and reduced LCAT concentration for CKD progression and renal function impairment in patients with and without diabetes.

**(A)**

|  |  |  |  |  |  | **95% C.I.** | |  |
| --- | --- | --- | --- | --- | --- | --- | --- | --- |
|  | **model** | **beta** | **S.E.** | **Wald F** | **HR** | **Lower** | **Upper** | **P** |
| Per 1 mg/dL in HDL-c reduction | 1 | 0.047 | 0.017 | 7.63 | 1.048 | 1.014 | 1.084 | 0.006 |
|  | 2 | 0.055 | 0.016 | 11.32 | 1.056 | 1.023 | 1.091 | 0.001 |
|  | 3 | 0.090 | 0.047 | 3.62 | 1.094 | 0.997 | 1.200 | 0.057 |
|  | 4 | 0.072 | 0.047 | 2.34 | 1.074 | 0.980 | 1.178 | 0.126 |
|  | | | | | | | | |
| Per 1 μg/mL reduction in LCAT concentration | 1 | 0.631 | 0.220 | 8.19 | 1.880 | 1.220 | 2.896 | 0.001 |
|  | 2 | 0.515 | 0.208 | 6.13 | 1.674 | 1.113 | 2.517 | 0.013 |
|  | 3 | 0.497 | 0.358 | 1.92 | 1.643 | 0.815 | 3.315 | 0.165 |
|  | 4 | 0.637 | 0.414 | 2.37 | 1.891 | 0.841 | 4.252 | 0.123 |

Tables report Hazard Risks (HR) (with lower and upper bounds of the 95% Confidence Interval (C.I.)) for dialysis entry and/or creatinine doubling per both one mg/dL reduction in HDL-c and ug/mL reduction in LCAT concentration in non-diabetic patients from the nefroPLIC cohort (among whom 22 CKD patients entered dialysis and/or doubled creatinine during follow-up). S.E.: standard error of the beta coefficient; Wald F: from Wald statistic for forward selection of co-variates (with entry testing (set at 0.05) and removal testing (set at 0.1)); *p*-value.

**(B)**

|  |  |  |  |  |  | **95% C.I.** | |  |
| --- | --- | --- | --- | --- | --- | --- | --- | --- |
|  | **model** | **beta** | **S.E.** | **Wald F** | **HR** | **Lower** | **Upper** | **P** |
| Per 1 mg/dL in HDL-c reduction | 1 | 0.059 | 0.029 | 4.17 | 1.061 | 1.002 | 1.123 | 0.041 |
|  | 2 | 0.063 | 0.034 | 3.47 | 1.065 | 0.997 | 1.138 | 0.062 |
|  | 3 | 0.129 | 0.051 | 6.39 | 1.138 | 1.029 | 1.258 | 0.011 |
|  | 4 | 0.118 | 0.054 | 4.88 | 1.126 | 1.013 | 1.250 | 0.027 |
|  | | | | | | | | |
| Per 1 μg/mL reduction in LCAT concentration | 1 | 0.370 | 0.268 | 1.91 | 1.448 | 0.857 | 2.447 | 0.166 |
|  | 2 | 0.501 | 0.297 | 2.84 | 1.650 | 0.922 | 2.951 | 0.092 |
|  | 3 | 1.175 | 0.627 | 3.52 | 3.240 | 0.949 | 11.062 | 0.061 |
|  | 4 | 2.396 | 1.216 | 3.88 | 10.980 | 1.012 | 119.164 | 0.049 |

Tables report Hazard Risks (HR) (with lower and upper bounds of the 95% Confidence Interval (C.I.)) for dialysis entry and/or creatinine doubling per both one mg/dL reduction in HDL-c and ug/mL reduction in LCAT concentration in diabetic patients from the nefroPLIC cohort (among whom 10 CKD patients entered dialysis and/or doubled creatinine during follow-up). S.E.: standard error of the beta coefficient; Wald F: from Wald statistic for forward selection of co-variates (with entry testing (set at 0.05) and removal testing (set at 0.1)); *p*-value.

**(C)**

|  |  |  | **95% C.I.** | |  |  |
| --- | --- | --- | --- | --- | --- | --- |
|  | **model** | **beta** | **Lower** | **Upper** | **t** | **P** |
| Per 1 mg/dL in HDL-c reduction | 1 | 0.015 | -0.018 | 0.040 | 0.884 | 0.378 |
|  | 2 | 0.014 | -0.019 | 0.048 | 0.844 | 0.400 |
|  | 3 | -0.007 | -0.044 | 0.031 | -0.357 | 0.722 |
|  | 4 | -0.007 | -0.046 | 0.033 | -0.332 | 0.541 |
| Per 1 μg/mL reduction in LCAT concentration | 1 | 0.835 | 0.408 | 1.263 | 3.86 | <0.001 |
|  | 2 | 0.783 | 0.366 | 1.199 | 3.71 | <0.001 |
|  | 3 | 0.731 | 0.307 | 1.154 | 3.41 | 0.001 |
|  | 4 | 0.734 | 0.282 | 1.185 | 3.21 | 0.002 |

Table reports annual reduction in eGFR both one mg/dL reduction in HDL-c and ug/mL reduction in LCAT concentration in the non-diabetic patients of the PLIC cohort. Beta coefficients are reported, with the lower and upper bounds around beta, the t-value (the size of the difference relative to the variation in HDL-c or LCAT concentration) and the *p*-value.

**(D)**

|  |  |  | **95% C.I.** | |  |  |
| --- | --- | --- | --- | --- | --- | --- |
|  | **model** | **beta** | **Lower** | **Upper** | **t** | **P** |
| Per 1 mg/dL in HDL-c reduction | 1 | 0.168 | 0.069 | 0.267 | 3.787 | 0.004 |
|  | 2 | 0.175 | -0.070 | 0.281 | 3.825 | 0.005 |
|  | 3 | -0.025 | -0.643 | 0.594 | -0.173 | 0.879 |
|  | 4 | -0.103 | -4.095 | 3.888 | -0.328 | 0.798 |
| Per 1 μg/mL reduction in LCAT concentration | 1 | 1.123 | -1.406 | 3.652 | 0.989 | 0.346 |
|  | 2 | 1.102 | -1.696 | 3.899 | 0.908 | 0.390 |
|  | 3 | 0.193 | -6.002 | 6.388 | 0.134 | 0.906 |
|  | 4 | 0.127 | -38.451 | 38.704 | 0.042 | 0.973 |

The table reports the annual reduction in eGFR, both the one mg/dL reduction in HDL-c and the ug/mL reduction in LCAT concentration in the diabetic patients of the PLIC cohort. Beta coefficients are reported, with the lower and upper bounds around beta, the t-value (the size of the difference relative to the variation in HDL-c or LCAT concentration) and the *p*-value.

For Table **(A)** and Table **(B)**: Model 1 is un-adjusted; Model 2 is adjusted by age and gender; Model 3 is adjusted by age, gender, BMI, systolic and diastolic blood pressure, albuminuria, total cholesterol, triglycerides, and glucose; Model 4 is adjusted by age, gender, BMI, systolic and diastolic blood pressure, anti-hypertensive treatments, albuminuria, total cholesterol, triglycerides, lipid lowering treatments, glucose, oral glucose lowering treatments.

For Table **(C)** and Table **(D)**: Model 1 is un-adjusted; Model 2 is adjusted by age and gender; Model 3 is adjusted by age, gender, BMI, systolic and diastolic blood pressure, total cholesterol, triglycerides, and glucose; Model 4 is adjusted by age, gender, BMI, systolic and diastolic blood pressure, anti-hypertensive treatments, total cholesterol, triglycerides, lipid lowering treatments, glucose, oral glucose lowering treatments.
